# Lack of LDL Receptor Enhances Amyloid Deposition and Decreases Glial Response in an Alzheimer's Disease Mouse Model

**DOI:** 10.1371/journal.pone.0021880

**Published:** 2011-07-06

**Authors:** Loukia Katsouri, Spiros Georgopoulos

**Affiliations:** Department of Cell Biology, Biomedical Research Foundation of the Academy of Athens, Athens, Greece; Virginia Commonwealth University, United States of America

## Abstract

**Background:**

Apolipoprotein E (ApoE), a cholesterol carrier associated with atherosclerosis, is a major risk factor for Alzheimer's disease (AD). The low-density lipoprotein receptor (LDLR) regulates ApoE levels in the periphery and in the central nervous system. LDLR has been identified on astrocytes and a number of studies show that it modulates amyloid deposition in AD transgenic mice. However these findings are controversial on whether LDLR deletion is beneficial or detrimental on the AD-like phenotype of the transgenic mice.

**Methodology/Principal Findings:**

To investigate the role of LDLR in the development of the amyloid related phenotype we used an APP/PS1 transgenic mouse (5XFAD) that develops an AD-like pathology with amyloid plaques, astrocytosis and microgliosis. We found that 4 months old 5XFAD transgenic mice on the LDLR deficient background (*LDLR-/-)* have increased amyloid plaque deposition. This increase is associated with a significant decrease in astrocytosis and microgliosis in the 5XFAD/*LDLR-/-* mice. To further elucidate the role of LDLR in relation with ApoE we have generated 5XFAD transgenic mice on the ApoE deficient (*ApoE-/-)* or the ApoE*/*LDLR double deficient background *(ApoE-/-/LDLR* -/-). We have found that ApoE deletion in the 4 months old 5XFAD/*ApoE*-/- mice decreases amyloid plaque formation as expected, but has no effect on astrocytosis or microgliosis. By comparison 5XFAD/*ApoE-/-LDLR -/-* double deficient mice of the same age have increased amyloid deposition with decreased astrocytosis and microgliosis.

**Conclusions:**

Our analysis shows that LDL deficiency regulates astrocytosis and microgliosis in an AD mouse model. This effect is independent of ApoE, as both 5XFAD/*LDLR -/-* and 5XFAD/*ApoE-/- LDLR -/-* mice show reduction in inflammatory response and increase in amyloid deposition compared to control mice. These results demonstrate that LDLR regulates glial response in this mouse model independently of ApoE and modifies amyloid deposition.

## Introduction

Alzheimer's disease (AD), the major form of dementia, is an age-related neurodegenerative disease that impairs basic cognitive functions, primarily memory. AD is characterized by age-dependant deposition of amyloid plaques and neurofibrillary tangles [1]. Apolipoprotein (ApoE) is the major susceptibility gene for the common late-onset form of Alzheimer's disease and the presence of the ε4 allele increases the risk of developing AD [2]. Accumulating evidence suggests that the differential effects of ApoE isoforms on Aβ aggregation and clearance play a major role in AD pathogenesis [3]. ApoE, a cholesterol carrier, is primarily synthesized in the liver and the central nervous system (CNS) [4]. ApoE within the CNS is synthesized by astrocytes and microglia [4]. Studies using huAPP transgenic mice that develop an AD-like phenotype have shown that ApoE deletion exerts a beneficial effect on Aβ-fibrillogenesis and amyloid plaque formation in the mouse brain without altering Aβ-production. APP transgenic mice deficient for ApoE show a dramatic decrease in fibrillary amyloid deposits [5], [6], [7], [8], [9]. ApoE binds to a number of membrane receptors, known as the low density lipoprotein receptor (LDLR) family. Many of these structurally related proteins, including the LDLR and the LDL receptor-related protein 1 (LRP1), have been shown to have diverse roles ranging from cholesterol homeostasis to nervous system development [10]. LDLR, the ancestor gene of the group, has been originally identified as a receptor for cholesterol-rich lipoproteins that regulates LDL-cholesterol metabolism [11]. LDLR deficiency in humans causes familial hypercholesterolemia (type IIA) [12] and inactivation of the LDLR gene in the mouse increases cholesterol levels and causes atherosclerosis [13].

Accumulating evidence suggests that cholesterol and cholesterol related proteins are involved in the pathogenesis of AD in humans and AD mouse models [14]. Although the LDLR has been shown to have a major role in cholesterol and ApoE homeostasis in the periphery [15], [16] its role in the CNS remains unclear [17]. Some LDLR polymorphisms showed a sex-dependent increased risk for developing AD in humans [18], [19]. In LDLR deficient mice, lack of LDLR increases brain ApoE but has no effect on hippocampal or CSF cholesterol [20]. A number of studies using AD mice have provided evidence that the family of proteins involved in cholesterol metabolism as the ABCA1, the ApoA1, the SR-BI and the LDLR are involved in the pathogenesis of the AD-like phenotype in the mouse brain [21], [22], [23], [24]. Previous studies using different AD mouse models that were LDLR deficient resulted in conflicting data on the effect of LDLR deficiency on the amyloid related phenotype of the mice [20], [25]. Lack of LDLR had no effect on amyloid deposition in the brain in a study using a huAPP transgenic mouse [20] while an analogous study using another huAPP transgenic mouse showed a significant increase in amyloid deposition [25]. A recent study where LDLR was over-expressed in the brains of APP transgenic mice resulted in a significant decrease of amyloid plaque formation, thus establishing an important role for LDLR in the amyloid related pathology in the mouse brain [26].

The focus of the present study was to elucidate the role of LDLR deficiency in the amyloid-related phenotype in an AD mouse model, as results from previous studies were conflicting, and to examine to what extent the effect on amyloid deposition caused by LDLR is exclusively mediated by ApoE. We used a transgenic mouse (5XFAD) that expresses huAPP and huPS1 mutant transgenes and develops an amyloid related pathology. Amyloid deposits first appear in the subiculum and spread to the hippocampus and the cortex. We found that 4 months old 5XFAD/*ApoE*-/- mice develop considerably less amyloid deposits, mainly located to the subiculum, compared to 5XFAD mice. We generated 5XFAD/*LDLR*-/-, 5XFAD/*ApoE*-/- and 5XFAD/*ApoE*-/-*LDLR*-/- double knock-out mice to evaluate the effect of the LDLR deficiency in the amyloid plaque formation on the *ApoE*+/+ and the *ApoE*-/- background. Thioflavine-S positive amyloid deposition was increased in the 5XFAD/*LDLR*-/- mice compared to the 5XFAD control mice as well as in the 5XFAD/*ApoE*-/-*LDLR*-/- compared to the 5XFAD/*ApoE*-/- mice. A significant decrease both in the astrocytic and microglial response was observed together with the increase in amyloid deposition in both the 5XFAD/*LDLR*-/- and 5XFAD/*ApoE*-/-*LDLR*-/- mice. These results demonstrate that the LDLR deficiency reduces the glial response in the 5XFAD mice and this effect is independent of ApoE.

## Results

### LDLR deficiency increases amyloid plaque formation both in the 5XFAD/*LDLR*-/- and the 5XFAD/*ApoE*-/-*LDLR*-/- transgenic mice

**Figure 1:**
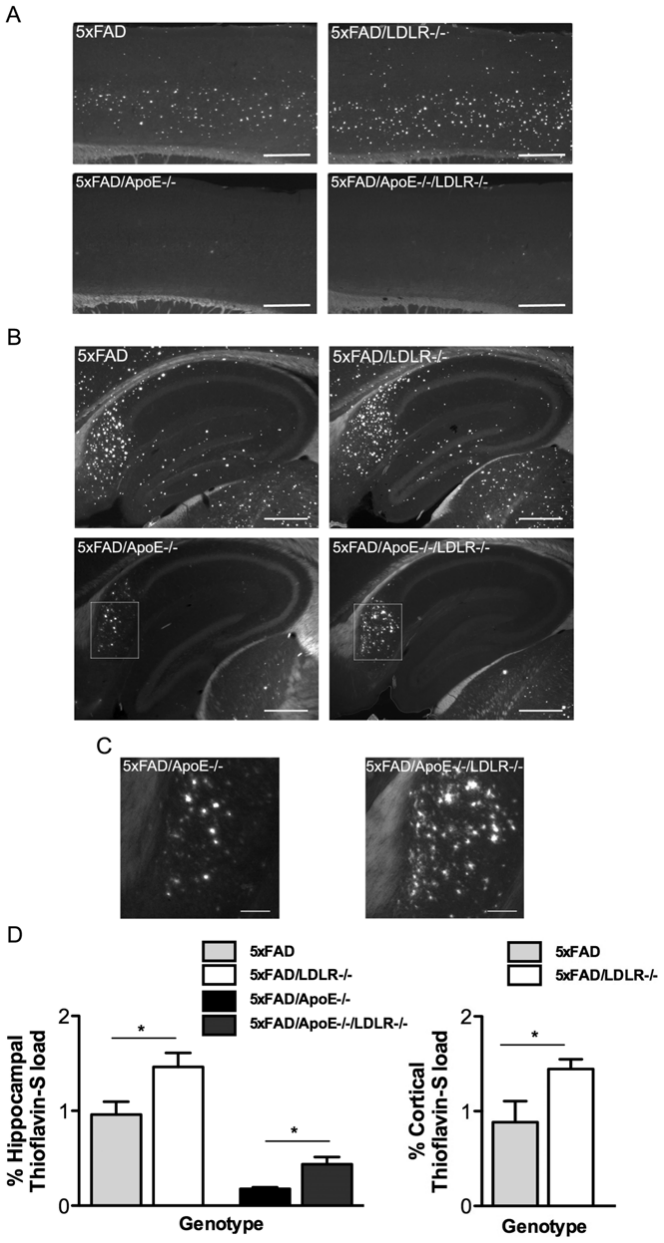
*LDLR* deficiency results in increased Thioflavine-S plaque load in the presence or in the absence of ApoE. A–B. Representative pictures of Thioflavine-S staining in cortices (A) and hippocampi (B) of the analyzed groups (n = 5−7, 6–7 sections per animal, 240 mm apart). The absence of LDLR results in increased Thioflavine-S positive staining in the 5XFAD mice in the presence (A and B upper photos) or the absence of ApoE (A and B lower photos). Scale bar 0.5 mm. C. Magnification of the subiculum of the 5XFAD/*ApoE*-/- and the 5XFAD/*ApoE*-/-*LDLR*-/- mice, showing the difference in abundance of Thioflavine-S positive plaques. Scale bar 0.1 mm. D. Quantitation of Thioflavine-S positive staining in the hippocampi (left) and cortices (right) of female mice showing the increase in the amyloid plaques in the 5XFAD/*LDL*-/- and 5XFAD/*ApoE*-/-*LDLR*-/- mice. One-way ANOVA showed a significant difference among groups (*P<0.0001)* followed by Student's t-test. * P<0.05. P-values among groups are analysed in Table 1.

To elucidate the effect of LDLR deficiency on the AD-like phenotype we used 4 months old female 5XFAD transgenic mice as male 5XFAD mice of the same age displayed a significant delay in the development of the amyloid pathology. A similar sex-specific effect on amyloid deposition has been reported on other AD mice [27]. Our analysis showed that 5XFAD/*LDLR*-/- mice showed a significant increase in Thioflavine-S positive amyloid deposits compared to the 5XFAD mice both in the hippocampus (Fig. 1B upper photos) and the cortex (Fig. 1A upper photos) (n = 7). Deletion of ApoE greatly reduced Thioflavine-S positive amyloid plaque formation in 5XFAD/*ApoE*-/- transgenic mice, with amyloid plaques restricted mainly at the subiculum (Fig. 1A and B lower photos). 5XFAD/*ApoE*-/-*LDLR*-/- double knock-out mice displayed a significant increase in amyloid plaque deposition, compared to the 5XFAD/*ApoE*-/- mice in the hippocampus (Fig. 1C) (n = 5−7). Quantification of the Thioflavine-S positive amyloid plaque load in the hippocampus and the cortex in these mice confirmed a significant increase in the absence of LDLR both in the presence and in the absence of ApoE (Fig. 1D). These data confirm that the absence of LDLR in the 5XFAD mouse model results in the increase of the amyloid plaque formation independently of ApoE.

**Table 1:** Results of Student's t-test for Thioflavine-S positive amyloid deposits of the analyzed groups.

Group 1 (hippocampus)	Group 2 (hippocampus)	P value
5xFAD	5xFAD/*LDLR*-/-	P = 0.03 (*)
5xFAD/*ApoE*-/-	5xFAD/*ApoE*-/-*LDLR*-/-	P = 0.0274 (*)
5xFAD	5xFAD/*ApoE*-/-	P = 0.013 (*)
5xFAD	5xFAD/*ApoE*-/-*LDLR*-/-	P = 0.0139 (*)
5xFAD/*LDLR*-/-	5xFAD/*ApoE*-/-	P = 0.0003 (***)
5xFAD/*LDLR*-/-	5xFAD/*ApoE*-/-*LDLR*-/-	P = 0.0003 (***)

Group 1 (Cortex)	Group 2 (Cortex)	P value
5xFAD	5xFAD/*LDLR*-/-	P = 0.0452 (*)

### LDLR deficiency increases ApoE in the brains of the 5XFAD/*LDLR*-/- mice and has no effect on APP processing

**Figure 2:**
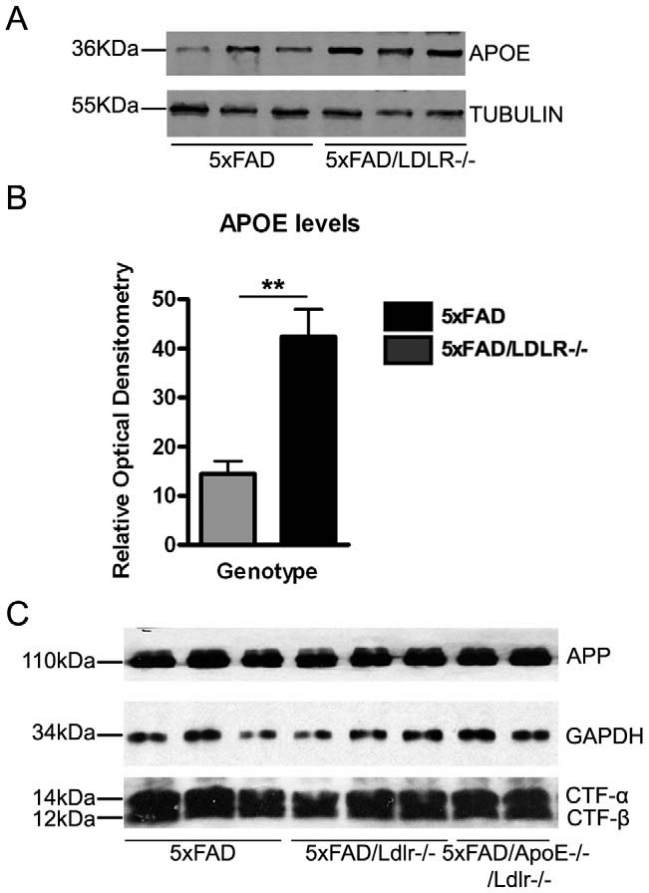
Lack of LDLR increases brain ApoE levels in the 5XFAD/*LDLR*-/- mice and has no effect on the APP processing. A. Western blot for ApoE of protein extracts of 5XFAD and 5XFAD/*LDLR*-/- mouse brains. Tubulin was used as a loading control. 30 µg from the guanidine fraction of brain homogenates was loaded in each lane. In the absence of LDLR the levels of brain ApoE are increased. B. Densitometry of western blots shows a significant increase in the levels of ApoE in the guanidine fraction of total brain extracts. Statistical analysis was performed with the Student's *t-test* (** P = 0.0041). C. 10 µg of protein from the lysis fraction of total brain homogenates were loaded in each lane and immunoblotted for full length APP and CTFs. GAPDH was used as a loading control. No difference was observed in the full length APP or the CTFs when normalised to the control indicating that LDLR deficiency has no effect on the steady-state levels of full-length APP or on α- and β-secretase activity (5-7 animals per genotype were analysed and the experiments were repeated 3 times).

To confirm the effect of LDLR deficiency on ApoE levels in the brains of the 5XFAD/*LDLR*-/- and 5XFAD control mice we extracted and immunoblotted the plaque-associated fraction from brain homogenates of the 5XFAD and 5XFAD/*LDL*-/- mice (Fig. 2A). We found a significant increase in the brain levels of ApoE in the 5XFAD/*LDLR* -/- mice compared to the 5XFAD mice (Fig. 2B), as it has been previously reported [20], [25].

To evaluate the effect of LDLR deletion on APP processing, we analyzed protein extracts from mouse brains. Western- blot analysis of total brain protein extracts for the full-length APP and its proteolytic fragments (α- and β-CTFs) did not detect any differences among the different genotypes (Fig. 2C). These data support that the LDLR deletion has no effect on the levels of APP or CTFs in the brains of the 5XFAD transgenic mice.

### LDLR deficiency increases Aβ deposition in the hippocampus of the 5XFAD/*LDLR*-/- and the 5XFAD/*ApoE*-/-*LDLR*-/-mice

**Figure 3:**
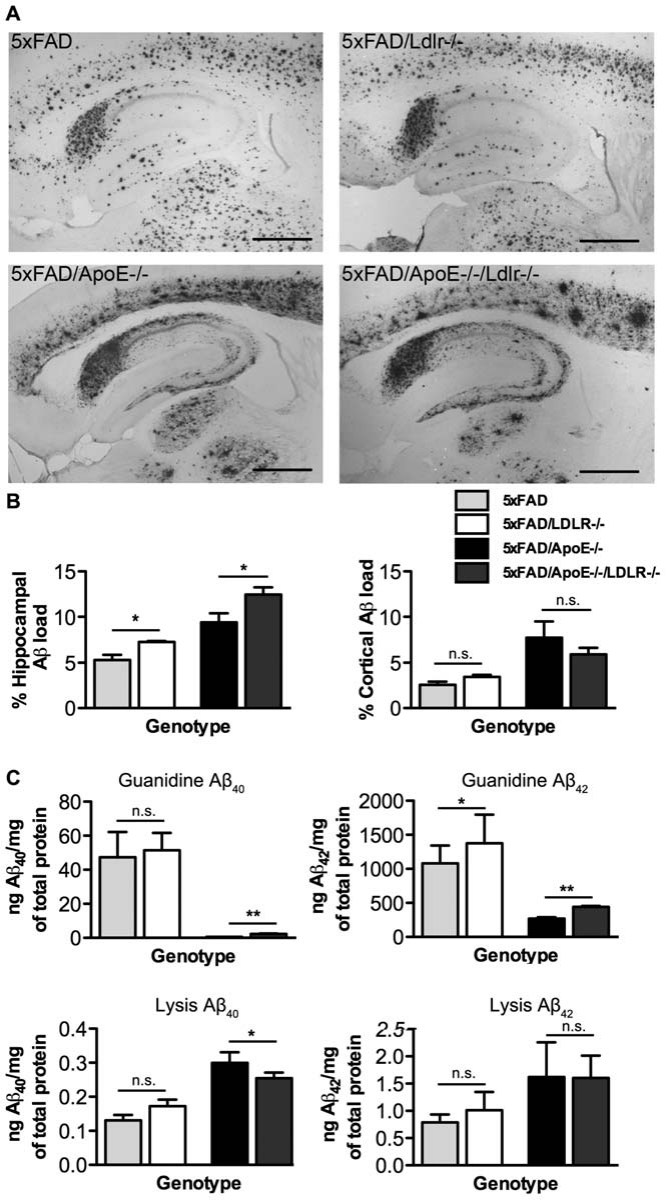
LDLR deletion increases Aβ deposition in the mouse hippocampus. A. Immunohistochemistry for total Aβ (6E10) in the brains of female mice. Representative pictures are shown for each genotype. The Aβ deposition is increased in the subiculum of the 5XFAD/*LDLR*-/- mice compared to the 5XFAD mice. The same effect is observed in the 5XFAD/*ApoE*-/-*LDLR*-/- mice where Aβ deposition is more intense in the subiculum. Aβ deposition in the 5XFAD/*ApoE*-/-*LDLR*-/- cortices appears more ‘dense’ and “compact” compared to the 5XFAD/*ApoE*-/- mice where Aβ deposition is more “diffuse”. Scale bar 0.5 mm (n = 5−7, 6–7 sections per animal, 240 mm apart). B. Quantitation of Aβ immunoreactivity in the hippocampi (left) of the analysed groups shows a significant increase in the Aβ deposition in the 5XFAD/*LDLR*-/- and the 5XFAD/*ApoE*-/-*LDLR*-/- mice. In the cortices (right) there is no significant difference between 5XFAD and 5XFAD/*LDLR*-/- as well as between 5XFAD/*ApoE*-/- and 5XFAD/*ApoE*-/-/*LDLR*-/-.*P<0.05. P-values among all groups are analysed in Table 2 and Table 3 for hippocampi and cortices respectively. C. Quantitation of Aβ_42_ and Aβ_40_ levels by ELISA in the 5XFAD and the 5XFAD/*LDLR*-/- mouse brain extracts showed an increase both in the guanidine and lysis fraction in the 5XFAD/*LDLR*-/- mice. A similar increase was also observed in the guanidine fraction of the 5XFAD/*ApoE*-/-*LDLR*-/- mice compared to the 5XFAD/*ApoE*-/- but not in the lysis fraction (n = 5−7) *P<0.05, **P<0.001.

Consistent with the increase in the Thioflavine-S positive amyloid deposits, 5XFAD/*LDLR*-/- and 5XFAD/*ApoE*-/-*LDLR*-/- transgenic mice displayed increased Aβ deposition detected by 6E10 antibody. Mouse brains of 4 months old 5XFAD/*LDLR*-/- mice had increased total Aβ immunoreactivity in the hippocampus and the cortex compared to 5XFAD control mice (Fig. 3A upper photos) (n = 7). This increase was statistically significant only in the hippocampus and not in the cortex (Fig. 3B). The pattern of Aβ-deposition in the 5XFAD and the 5XFAD/*LDLR*-/- mice was similar with more intense deposition in the subiculum and in the molecular layer of the dentate gyrus of the hippocampus. A significant increase in Aβ immunoreactivity was also noticed in the hippocampus of the 5XFAD/*ApoE*-/-*LDLR*-/- mice compared to the 5XFAD/*ApoE*-/- mice (n = 5−7) (Fig. 3A lower photos and 3B left graph). Also the pattern of Aβ-deposition was similar with a characteristic lack of deposition in the molecular layer of the dentate gyrus of the hippocampus both in the 5XFAD/*ApoE*-/- and the 5XFAD/*ApoE*-/-*LDLR*-/- mice. Comparison of Aβ-deposition in the cortices of the 5XFAD/*ApoE*-/- and the 5XFAD/*ApoE*-/-*LDLR*-/- mice showed a non significant increase in the 5XFAD/*ApoE*-/- brains (Fig. 3B right graph). This could be explained by the different pattern of Aβ deposits between the two genotypes in the cortex. 5XFAD/*ApoE*-/- mice developed a more ‘diffuse’ pattern covering a larger area, compared to the 5XFAD/*ApoE*-/-*LDLR*-/- mice that showed a more ‘compact’ pattern covering a smaller area (Fig. 3A lower photos).

**Table 2:** Results of Student's t-test for Aβ immunostaining in the hippocampi of the analyzed groups.

Group 1	Group 2	P value
5XFAD	5XFAD/*LDLR*-/-	P = 0.0281 (*)
5XFAD/*ApoE*-/-	5XFAD/*ApoE*-/-*LDLR*-/-	P = 0.0493 (*)
5XFAD	5XFAD/*ApoE*-/-	P = 0.0059 (**)
5XFAD	5XFAD/*ApoE*-/-*LDLR*-/-	P = 0.0001 (***)
5XFAD/*LDLR*-/-	5XFAD/*ApoE*-/-	P = 0.0215 (*)
5XFAD/*LDLR*-/-	5XFAD/*ApoE*-/-*LDLR*-/-	P<0.0001 (***)

**Table 3:** Results of Student's t-test for Aβ immunostaining in the cortices of the analyzed groups.

Group 1	Group 2	P value
5XFAD	5XFAD/*LDLR*-/-	P = 0.0546 (ns)
5XFAD/*ApoE*-/-	5XFAD/ApoE-/-*LDLR*-/-	P = 0.3385 (ns)
5XFAD	5XFAD/*ApoE*-/-	P = 0.007 (**)
5XFAD	5XFAD/*ApoE*-/-*LDLR*-/-	P = 0.0297 (*)
5XFAD/*LDLR*-/-	5XFAD/*ApoE*-/-	P = 0.0179 (*)
5XFAD/*LDLR*-/-	5XFAD/*ApoE*-/-*LDLR*-/-	P = 0.006 (**)

Analysis of Aβ_40_ and Aβ_42_ levels by ELISA between 5XFAD and 5XFAD*/LDLR*-*/-* mice showed an increase both in the guanidine and lysis fractions in the 5XFAD*/LDLR*-*/-* brains, reflecting the increase in Thioflavine-S deposition observed in the 5XFAD*/LDLR*-*/-* mice. The same analysis between the 5XFAD*/ApoE-/-* and the 5XFAD/*ApoE-/-LDLR-/-* mice showed an increase in both Aβ_40_ and Aβ_42_ levels in the guanidine extract.

This increase of Aβ_40_ and Aβ_42_ levels observed mainly in the guanidine fraction suggests that LDLR deficiency promotes fibrillar Aβ formation (Fig. 3C.)

### 5XFAD/*LDLR*-/- and 5XFAD/*ApoE*-/-*LDLR*-/- transgenic mice display decreased astrocytic and microglial response

**Figure 4:**
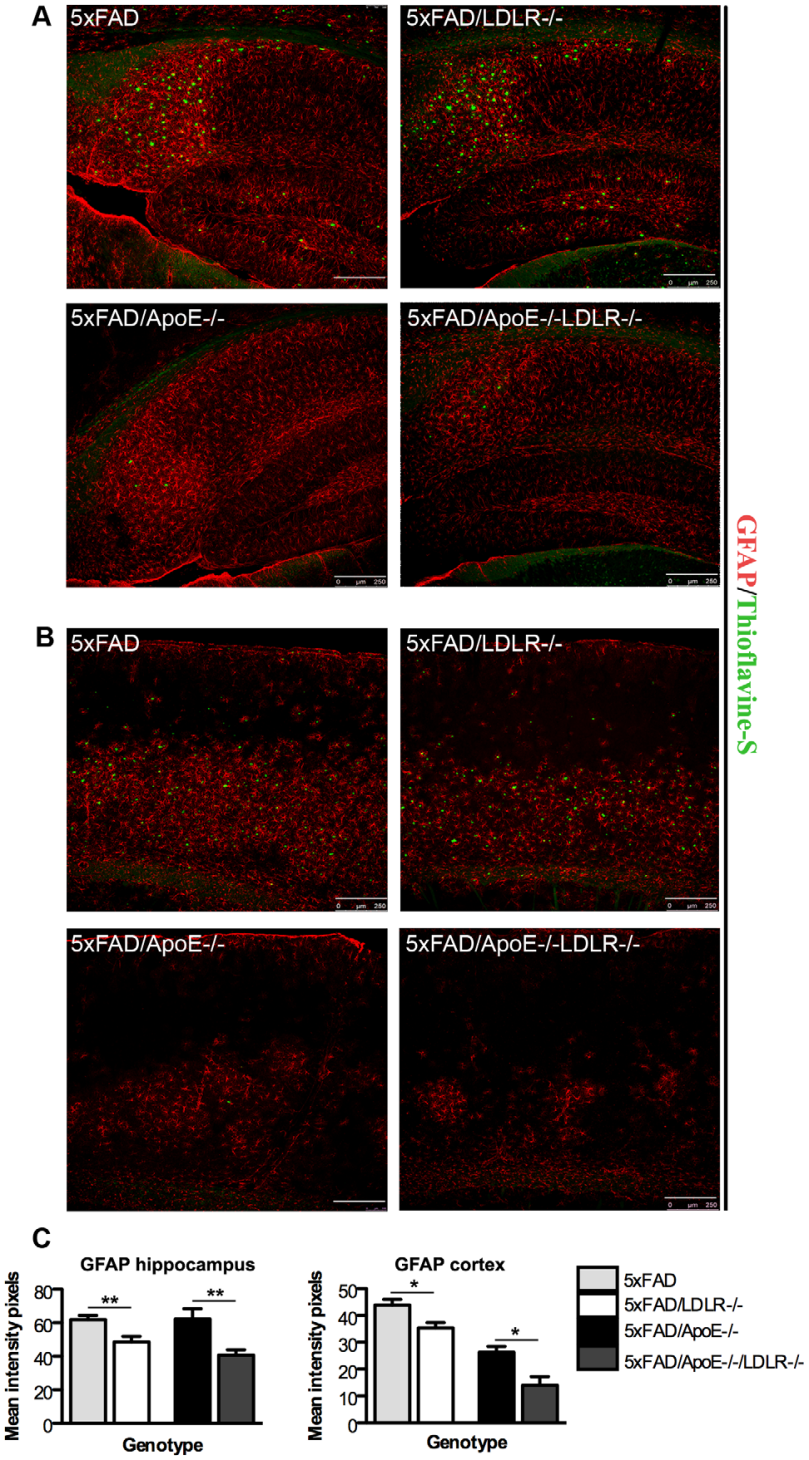
Astrocytosis is decreased in the brains of 5XFAD/*LDLR*-/- and 5XFAD/*ApoE-/- LDLR*-/- mice. Immunohistochemistry for GFAP (red) and Thioflavine-S (green) in the hippocampi (A) and the cortices (B) of mouse brains. Representative pictures are shown for each genotype. In both areas examined the absence of LDLR results in reduction of GFAP immunoreactivity. Astrocytosis in both the cortex and the hippocampus is more intense in the 5XFAD and the 5XFAD/*ApoE*-/- compared to the 5XFAD/*LDLR*-/- and the 5XFAD/*ApoE*-/-*LDLR*-/- sections respectively. Scale bar 250 µm. The pictures were taken under the same conditions of intensity and the experiment was repeated 3 times. (n = 5−7, 3−4 independent sections per animal were analyzed). C. Quantitation of GFAP positive staining in the hippocampi and cortices showing a decrease in the 5XFAD/*LDL*-/- and 5XFAD/*ApoE*-/-*LDLR*-/- mice compared to the control mice. P-values in the hippocampi (left graph) ** P = 0.0040 for 5XFAD vs. 5XFAD/LDLR-/- and P = 0.0031 for 5XFAD/ApoE-/- vs. 5XFAD/ApoE-/-LDLR-/-. In the cortices (right graph) * P = 0.0399 for 5XFAD vs. 5XFAD/LDLR-/- and P = 0.0205 for 5XFAD/ApoE-/- vs. 5XFAD/ApoE-/-LDLR-/-.

**Figure 5:**
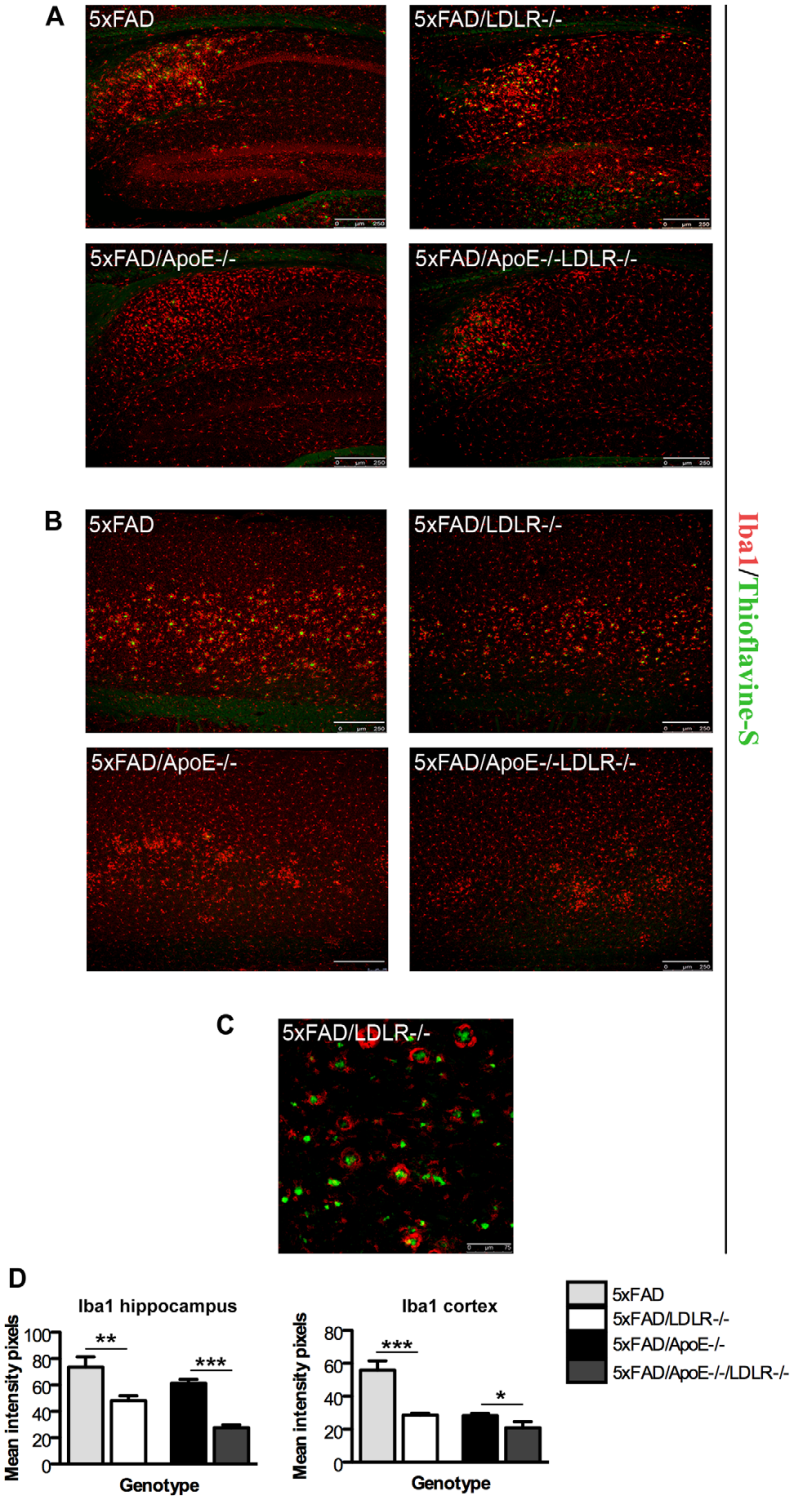
Microgliosis is decreased in the brains of 5XFAD/*LDLR*-/- and 5XFAD/*ApoE-/-LDLR*-/- mice. A. Immunohistochemistry for Iba1 (red) and Thioflavine-S (green) in the hippocampus and cortex of mouse brains. Representative pictures are shown for each genotype. *LDLR* deficiency results in the reduction of microgliosis both in the hippocampi (A) and the cortices (B) in 5XFAD/*LDLR*-/- and 5XFAD/ *ApoE-/- LDLR*-/- mice. C. Iba1 positive round cell formations surrounding Thioflavine-S positive amyloid deposits in the thalamus of 5XFAD/LDLR-/- mice. Scale bar 75 µm. The pictures were taken under the same conditions of intensity and the experiment was repeated 3 times. (n = 5-7, 3-4 independent sections per animal were analyzed). D. Quantitation of Iba1 positive staining in the hippocampi and cortices showing a decrease in the 5XFAD/*LDL*-/- and 5XFAD/*ApoE*-/-*LDLR*-/- mice compared to the control mice. P-values in the hippocampi (left graph) ** P = 0.0043 for 5XFAD vs. 5XFAD/LDLR-/- and ***P<0.0001 for 5XFAD/ApoE-/- vs. 5XFAD/ApoE-/-LDLR-/-. In the cortices (right graph) *** P<0.0001 for 5XFAD vs. 5XFAD/LDLR-/- and *P = 0.0487 for 5XFAD/ApoE-/- vs. 5XFAD/ApoE-/-LDLR-/-.

5XFAD mice have been shown to have extended neuroinflammatory responses displaying a significant increase in astrocytic and microglial response that is associated with age and amyloid deposition [28]. To evaluate the effect of LDLR deficiency on the glial response, astrocytic and microglial, we analysed female 4 month old 5XFAD and 5XFAD/*LDLR*-/- mice as well as 5XFAD/*ApoE*-/- and 5XFAD/ *ApoE*-/-*LDLR*-/- mice. Despite the increase in the amyloid deposition that was expected to further increase the glial response, in the 5XFAD/*LDLR*-/- mice GFAP (astrocytic) and Iba-1 (microglial) staining was reduced compared to 5XFAD littermates of the same gender and age. A similar effect was exerted in the 5XFAD/*ApoE*-/-*LDLR*-/- mice where astrocytic and microglial response was reduced compared to the 5XFAD/*ApoE*-/- (Figs. 4A,B,C and 5A,B,C). This decrease of GFAP and Iba1 staining was localized in the subiculum of the hippocampus (Figs. 4A and 5A) as well as in the cortex where it was more profound (Figs. 4B and 5B). In the thalamus of the 5XFAD/*LDLR*-/- mice we also noticed the formation of Iba-1 positive round cell formations surrounding Thioflavine-S positive amyloid deposits not observed in any other genotype (Fig. 5C).

These results indicate that the LDLR deficiency is affecting the neuroinflammatory response in the 5XFAD mouse model. Our results also suggest that this effect is not mediated by ApoE.

## Discussion

ApoE is a major risk factor in the pathogenesis of AD in the human population. The ε4 allele of ApoE predisposes to AD while the ε2 allele is protective [29], [30]. ApoE2 has been shown to have a very low binding capacity to the LDLR compared to ApoE3 or ApoE4 [4]. This difference in the binding ability to LDLR has lead to the hypothesis that LDLR is a potential mediator of the differential effect of the ApoE alleles on Aβ clearance and aggregation. Previous studies where the LDLR gene was deleted or over-expressed in AD transgenic mice indeed confirmed the role of the LDLR in amyloid deposition [20], [25], [27]. In all these studies LDLR was shown to regulate ApoE levels in the brain suggesting that ApoE is possibly the key mediator of the effect on amyloid deposition. Nevertheless deletion of LDLR in huAPP transgenic mice into two separate studies resulted in opposing evidence on the effect of LDLR deletion in amyloid deposition, with one study having no effect and the other showing a significant increase [20], [25]. To clarify the role of LDLR deficiency in amyloid deposition in a huAPP transgenic mouse model we generated and analyzed a novel huAPP/PS1 transgenic mouse deficient for LDLR. Also in the present study we examined whether LDLR effect on amyloid deposition is dependent on ApoE, or whether LDLR can exert any effect independent of ApoE on the pathogenesis of the amyloid related phenotype in this AD mouse model. ApoE has a major impact on the generation of amyloid deposits in the AD mouse brain and deletion of ApoE in most AD mouse models significantly inhibits amyloid plaque formation [6], [7], [8]. In this study we used an AD mouse model (5XFAD) that carries both huAPP and huPS1 mutant transgenes and starts to develop amyloid deposits at the early age of two months [28]. We backcrossed 5XFAD transgenic mice to the ApoE deficient background and we found a great reduction in amyloid load as expected. Although there was a significant reduction in amyloid deposition, 5XFAD/*ApoE*-/- mice developed amyloid plaques in the subiculum at the early age of four months. Also as it has been reported with other APP/PS1 transgenic mice, we observed a sex-specific effect on amyloid deposition with 5XFAD female mice developing amyloid deposits earlier compared to male littermates [27], [31], [32]. As the prevalence of AD is higher in women it is noteworthy that a similar mechanism is likely to be present in the mice [33].

To evaluate the effect of LDLR deletion on amyloid deposition we generated 5XFAD/*LDLR*-/- mice and compared amyloid deposition to the 5XFAD mice. We found that LDLR deletion increases Thioflavine-S positive amyloid deposits both in the hippocampus and the cortex as it has been previously reported using a different AD mouse model. [25]. We also found that ApoE levels are increased in the 5XFAD/*LDLR*-/- mice compared to 5XFAD mice suggesting that LDLR regulates ApoE levels in the brain.

Next, to examine whether the increase in amyloid deposition induced by the LDLR deficiency is mediated exclusively by the effect of LDLR on ApoE, we generated 5XFAD/*ApoE*-/-*LDLR*-/- mice and compared amyloid deposition to the 5XFAD/*ApoE*-/- mice. We found that deletion of LDLR in the 5XFAD/*ApoE*-/-*LDLR*-/- mice increased amyloid deposition in the absence of ApoE. The pattern of formation of amyloid deposits both in the 5XFAD/*ApoE*-/- and the 5XFAD/*ApoE*-/-*LDLR*-/- mice was characteristic with absence of deposits in the molecular layer of the hillus of the hippocampus suggesting that lack of LDLR can enhance amyloid deposition but cannot modify the pattern of deposition that is dictated by ApoE. We also found that LDL deletion or double ApoE LDLR deletion has no impact on the APP processing and production of α and β-CTFs fragments in the mouse brain, suggesting that the effect on amyloid deposition resulted from increased Aβ aggregation and reduced clearance. Our results, using the 5XFAD mouse model, confirm that LDLR is involved in amyloid deposition as shown in previous studies. Deletion of LDLR confers a negative effect according to our data and other studies [20], [25] while over-expression diminishes amyloid deposition [27]. Moreover our data demonstrate that LDLR deletion has an effect on amyloid deposition independent of ApoE as 5XFAD/*ApoE*-/-*LDLR*-/- mice have increased amyloid deposition compared to the 5XFAD/*ApoE*-/- mice. These results imply that LDLR might be involved in a mechanism affecting amyloid deposition where ApoE is not involved.

A major characteristic of AD pathology is the increased inflammatory response that is observed close and around the amyloid plaques. Many studies have suggested that the brain's immune system, microglia and astrocytes play a major role in AD pathogenesis [34], [35]. As 5XFAD transgenic mice display early astrogliosis and microgliosis associated with amyloid deposits, we examined the effect of LDLR deficiency on astrogliosis and microgliosis in the mouse brain. We found that despite the increase of amyloid deposits in the 5XFAD/*LDLR*-/- mice there was a profound decrease both in the astrocytic and microglial response compared to the 5XFAD mice. The same effect was observed in the 5XFAD/*ApoE*-/-*LDLR*-/- mice compared to the 5XFAD/*ApoE*-/- mice. This was very obvious in the subiculum and in the cortex where amyloid plaques were formed. This was not expected as microglial and astrocytic response is proportional to the amyloid deposition and was expected to be more than less compared to the controls. These data clearly suggest for the possible involvement of LDLR in the glial response in the AD mouse brain. Also this effect is not related to ApoE as it is observed both in the 5XFAD/*LDL*-/- and the 5XFAD/*ApoE*-/-*LDLR*-/- mice. An effect in the glial response related to LDLR has been also reported in another study where an LDLR transgene has been expressed in the brain of another AD mouse model [27]. In this study LDLR over-expression resulted in reduced amyloid deposition with decreased glial response. Glial response has been shown to be proportional to the amyloid deposition in most transgenic mice therefore a reduction in astrocytic and microglial response is an expected effect following reduced amyloid deposition.

Our study suggests that LDLR is not only able to regulate ApoE levels in the brain thus affecting amyloid deposition, but it can have an effect on amyloid deposition independent of ApoE by regulating glial response. Our data along with previous studies show that LDLR has an effect on amyloid deposition as other members of the LDLR family of receptors. LRP1 has also been shown to be involved in the clearance of amyloid [36], [37], [38]. It is likely that the LDLR acts jointly with the LRP1 and the deletion of LDLR is partially compensated by LRP1 in the clearance of Aβ.

In this study we also showed that LDLR is able to regulate the glial response, both astrocytic and microglial in the absence of ApoE. The increased formation of amyloid deposits in the 5XFAD/*ApoE*-/-*LDLR*-/- mice can be attributed to down-regulation of the glial inflammatory response. A defective cholesterol homeostasis can be a possible explanation for this effect. Abnormal cholesterol homeostasis caused by deletion of ApoE or other cholesterol receptors as the SR-BI has been shown to cause altered responses to different immune cell types like the macrophages [39], although LDLR has not been implicated in regulating immune responses in the brain or the periphery so far.

The present study further implicates LDLR in the pathogenesis of AD suggesting LDLR as a potential regulator of the glial response in the development of the AD-like phenotype in an AD mouse model. Moreover we show that this effect can be independent of ApoE suggesting a novel mechanism.

In conclusion, our results provide new evidence regarding the role of LDLR in the pathogenesis of Alzheimer's disease suggesting that LDLR could be a potential therapeutical target in AD.

## Materials and Methods

### Animals

*LDLR*-/-, *ApoE*-/- and 5XFAD transgenic mice were obtained from The Jackson Laboratory, Bar Harbor, ME. *ApoE*-/- and 5XFAD mice were on the C57Bl6/J background. LDLR-/- were on C57Bl6/J;129S2 mixed genetic background and have been backcrossed for at least six generations to C57Bl6/J background. All test and control mouse groups analyzed for Aβ related pathology were 4 months old. Only female mice were analyzed as female 5XFAD transgenic mice develop the amyloid related phenotype earlier compared to males of the same age. To generate 5XFAD/*LDLR*-/- mice, 5XFAD mice were mated with *LDLR*-/- mice and the resulting 5XFAD/*LDLR*+/- mice were mated with the *LDLR*-/- mice. 5XFAD/*LDLR*-/- were identified by PCR. The same strategy was used to generate 5XFAD/*ApoE*-/- mice. 5XFAD mice were mated with *ApoE*-/- mice and the resulting 5XFAD/*ApoE*+/- mice were mated with the *ApoE*-/- mice. 5XFAD/*ApoE*-/- mice were identified by PCR. To generate *ApoE*-/-*LDLR*-/- mice, ApoE-/- mice were mated to *LDLR*-/- mice. The *ApoE*+/-*LDLR*+/- that resulted from this cross were mated with each other and *ApoE*-/-LDLR-/- mice were identified by PCR. To generate 5XFAD/*ApoE*-/-*LDLR*-/- mice 5XFAD mice were mated with *ApoE*-/-*LDLR*-/- mice and the resulting 5XFAD/*ApoE*+/-*LDLR*+/- mice were mated with the *ApoE*-/-*LDLR*-/- mice. 5XFAD/*ApoE*-/-*LDLR*-/- mice were identified by PCR. All mice were maintained on a standard chow diet containing 5% fat. All animal procedures were approved by the Bioethical committee of the Biomedical Research Institute of the Academy of Athens and by the Prefecture of Athens, Greece. All animal experimentations were carried out in agreement with ethical recommendation of the European Communities Council Directive of 24 November 1986 (86/609/EEC). Standard PCR techniques were used to genotype the 5XFAD, ApoE and LDLR genotypes.

### Tissue collection

Mice were anesthetized and transcardially perfused with PBS 0.1 M pH 7.4. Tissues were harvested immediately after perfusion. Left hemibrains were snap-frozen in liquid nitrogen for protein analysis. Right hemibrains were immersion-fixed in 4% PFA in PBS for 48 hours and cryoprotected in 20% sucrose in PBS for immunohistochemistry.

### Protein extraction from brain tissue

Protein extraction from left hemibrains was performed in three consecutive steps in order to evaluate protein levels in PBS, Lysis buffer (containing 10% glycerol, 1% Triton X-100 and complete protease inhibitor (Roche Applied Science) in PBS) and Guanidine fractions (5 M Guanidine-HCl) respectively as described before [21]. Brain tissue from all animals was extracted in an identical manner, and all fractions were immediately frozen at -80°C until analysis. Protein concentrations were determined by BCA Protein Assay (Pierce).

### Western blot

Left hemibrains tissues were homogenised as designated above and 30 µg of total protein were separated in a 10% SDS-PAGE Tris-Glycine for ApoE and Tubulin. In brief, after electrophoresis, proteins were transferred to a PVDF membrane (Immobilon-P, Millipore), blocked for 1 hour at room temperature with 5% milk in TBS-Tween 0.05% (TBST) and incubated for 1 hour at room temperature with goat anti-ApoE (M20, 1∶2000, Santa Cruz) or mouse anti-Tubulin (3D10, 1∶500, Sigma) in blocking solution. After washes with TBST blots were incubated with appropriate secondary antibodies (Santa Cruz) for 1 hour. For APP western blot 10 µg of total protein were separated in a 12% Tris-Tricine gel and transferred to nitrocellulose membrane (Protran, Whatman). Membranes were blocked for 30 minutes at 37°C in 5% FCS and incubated overnight at 4°C with rabbit polyclonal anti-human APP 1∶2500 [R1(57), a kind gift by Dr. P. Mehta, Institute for basic research in developmental disabilities, Staten Island, NY] [40]. The following day the membrane was washed 3 times with TBST and incubated for 1 hour at room temperature with goat-anti-rabbit HRP-conjugated (Santa Cruz). Blots were developed using ECL (Amersham) according to the manufacturer's recommendations. Bands were quantitated by densitometry using NIH Image J software.

### Immunohistochemistry

Brains were fixed in 4% PFA in PBS for 48 hrs and then were cryoprotected in 20% sucrose in PBS (0.1 M PBS, pH 7.4). Fixed hemibrains were cut in 40-µm sagittal floating sections from the genu of the corpus callosum to the most caudal hippocampus using a vibratome (Leica VT1000S). For total Aβ immunostaining 6–7 sagittal sections 240 µm apart from each other spanning all the hippocampal formation were chosen. Sections were washed with TBS-0.1% Triton X-100 3 times for 10 minutes each, incubated for 30 min with 0.3% H_2_O_2_ in TBS and washed with TBS 3 times. Antigen retrieval was performed with 98% formic acid for 5 min, followed by 3 washes with TBS. Sections were transferred into blocking solution containing 15% normal donkey serum (Vector Laboratories) in TBS-Triton X-100 0.1% for 1 hour and incubated overnight at 4°C in 5% normal donkey serum (Vector Laboratories) in TBS-Triton X-100 0.1% containing the monoclonal biotinylated 6E10 primary antibody (1∶1000, Signet). Sections were then washed five times with TBS-Triton X-100 0.1%, and incubation in avidin-biotinylated horseradish peroxidase complex (ABC Elite, Vector Laboratories) followed for 90 min at room temperature. Peroxidase labelling was visualized with DAB/Ni (Vector). After a 1-min incubation period, sections were washed, mounted on glass slides, dehydrated in increasing ethanol concentrations from 50 to 100% followed by xylene and coverslipped with DPX (BDH).

The specificity of immunoreactivity was confirmed both by omitting the primary antibody and by the lack of signal when applying the same protocol on brain sections of wild-type littermates.

### Immunofluorescence

For the estimation of amyloid plaque load in transgenic mice, 6–7 sagittal sections 240 µm apart from each other spanning all the hippocampal formation were chosen for Thioflavine-S staining. Sections were incubated for 8 minutes in aqueous solution of Thioflavine-S (1% w/v) and then differentiated 2 times with 80% Ethanol for 3 minutes each, followed by another 3-minutes wash with 95% Ethanol. Sections were rinsed three times with ddH_2_O and coverslipped with Vectashield fluorescence mounting medium (Vector). Imaging for Thioflavine-S was performed on a Leica DMRA 2 microscope (magnification 2.5x for cortices and 5x for hippocampi) and image captivation was performed using Leica Application Suite, version 2.8.1 software. For the analysis of the inflammatory response in the brains of mice, the GFAP and Iba1 markers for astrocytes and microglia respectively were used. For GFAP and Thioflavine-S, free-floating sections were washed 3 times with 1×TBS pH 7.6, blocked with 1×TBS-Triton X-100 0.4%, 5% Normal Goat Serum (Vector) for 1 hour at room temperature and incubated overnight at 4°C with rabbit polyclonal anti-GFAP (1∶1000, Sigma). The following day, sections were washed five times with 1×TBS and incubated in blocking solution with goat anti-rabbit Cy3-conjugated (1∶300, Jackson ImmunoResearch) for 1 hour at room temperature. Sections were washed with 1×TBS and immersed in Thioflavine-S (1% w/v aqueous solution) for 5 minutes. Sections were differentiated twice with 70% Ethanol and rinsed 3 times with PBS (0,1 M pH 7.4). Sections were mounted on glass slides and coversliped with Vectashield fluorescence mounting medium (Vector). For Iba1 and Thioflavine-S immunofluorescence sections were permeabilized for 30 minutes with PBS-Triton X-100 0.1% at room temperature, blocked for 3 hours with 10% fetal calf serum (FCS), 1% BSA (fraction V, Sigma) in PBS-Triton X-100 0.1% and incubated overnight at 4°C with rabbit anti-Iba1 (1∶300, Wako) in 1% FCS, 0.1% BSA, PBS-Triton X-100 0.01%. The following day, sections were washed 5 times with PBS and incubated with goat anti-rabbit Cy3-conjugated (1∶300, Jackson Immunoresearch) for 1 hour at room temperature. Sections were washed 5 times with 1×TBS and stained for Thioflavine-S as described above. Imaging was performed with a Leica TCS SP5 Confocal microscope and pictures were captured and analyzed with the Leica LAS AF Suite.

### Quantitation and statistical analysis

Images were analysed with the NIH Image J software. For the Aβ or Thioflavine-S load the images were thresholded within a linear range and the load was defined as the per cent area covered by Aβ or Thioflavin-S positive plaques (% Aβ or Thioflavin-S load). For Iba1 and GFAP the mean intensity of the immunofluorescent signal within a linear range was analysed. All data are expressed as mean±SEM. Statistical significance of differences was evaluated either with Student's *t*-test or with one way ANOVA followed by the two-way Student's *t*-test. In the *t*-test analyses, Welch's correction for unequal variances was applied when variances were significantly different between groups. Probability values *P<0.05* were considered significant. All statistical analyses were performed using GraphPad Prism (version 4.0; Graphpad software for Science Inc., San Diego).
